# A reference genome sequence for the exceptionally long-lived Great Basin bristlecone pine, *Pinus longaeva*

**DOI:** 10.1093/g3journal/jkag064

**Published:** 2026-03-17

**Authors:** David B Neale, Aleksey V Zimin, Constance I Millar, Patrick E McGuire, Jessica A Hosea, Edward Li, Daniela Puiu, Winston Timp, Steven L Salzberg

**Affiliations:** Department of Plant Sciences, University of California Davis, Davis, CA 95616, United States; Department of Biomedical Engineering and Center for Computational Biology, Johns Hopkins University, Baltimore, MD 21218, United States; USDA Forest Service, Pacific Southwest Research Station, Vallejo, CA 94592, United States; Department of Plant Sciences, University of California Davis, Davis, CA 95616, United States; Department of Biomedical Engineering and Center for Computational Biology, Johns Hopkins University, Baltimore, MD 21218, United States; Department of Biomedical Engineering and Center for Computational Biology, Johns Hopkins University, Baltimore, MD 21218, United States; Department of Biomedical Engineering and Center for Computational Biology, Johns Hopkins University, Baltimore, MD 21218, United States; Department of Biomedical Engineering and Center for Computational Biology, Johns Hopkins University, Baltimore, MD 21218, United States; Department of Biomedical Engineering and Center for Computational Biology, Johns Hopkins University, Baltimore, MD 21218, United States; Department of Computer Science, Johns Hopkins University, Baltimore, MD 21218, United States

**Keywords:** *Pinus longaeva*, bristlecone pine, conifer, genome assembly, annotation, NLR, telomere length

## Abstract

Great Basin bristlecone pine (*Pinus longaeva*), one of two species of bristlecone pine, the other being Rocky Mountain bristlecone pine (*P. aristata*), is endemic to the high Great Basin mountains in eastern California, Nevada, and Utah. It is the upper treeline forest tree in this region, found mostly between 2900 and 3600 m. The primary goal of this project was to generate a reference genome sequence for *P. longaeva* that, among its many possible applications, will serve as an important genetic resource to better understand the genetic mechanisms underlying its extreme longevity and its adaptation to the extreme environmental conditions where it is found. A combination of short-read and long-read sequences were generated from haploid megagametophyte and diploid needle tissues, respectively. A customized genome assembly approach was used to construct a highly contiguous 23.8-gigabase genome with a scaffold N50 size of 1.2 gigabases. The chloroplast and mitochondrial genomes were assembled separately into circular chromosomes with lengths of 120 kilobases and 8.68 megabases, respectively. While the number of disease resistance genes known as nucleotide-binding leucine-rich repeat receptors (NLRs) and larger-than-average telomere lengths relative to other conifers have been suggested as genetic mechanisms for controlling longevity, we did not find strong evidence for their involvement. Clearly further study is needed.

## Introduction

**Fig. 1.**
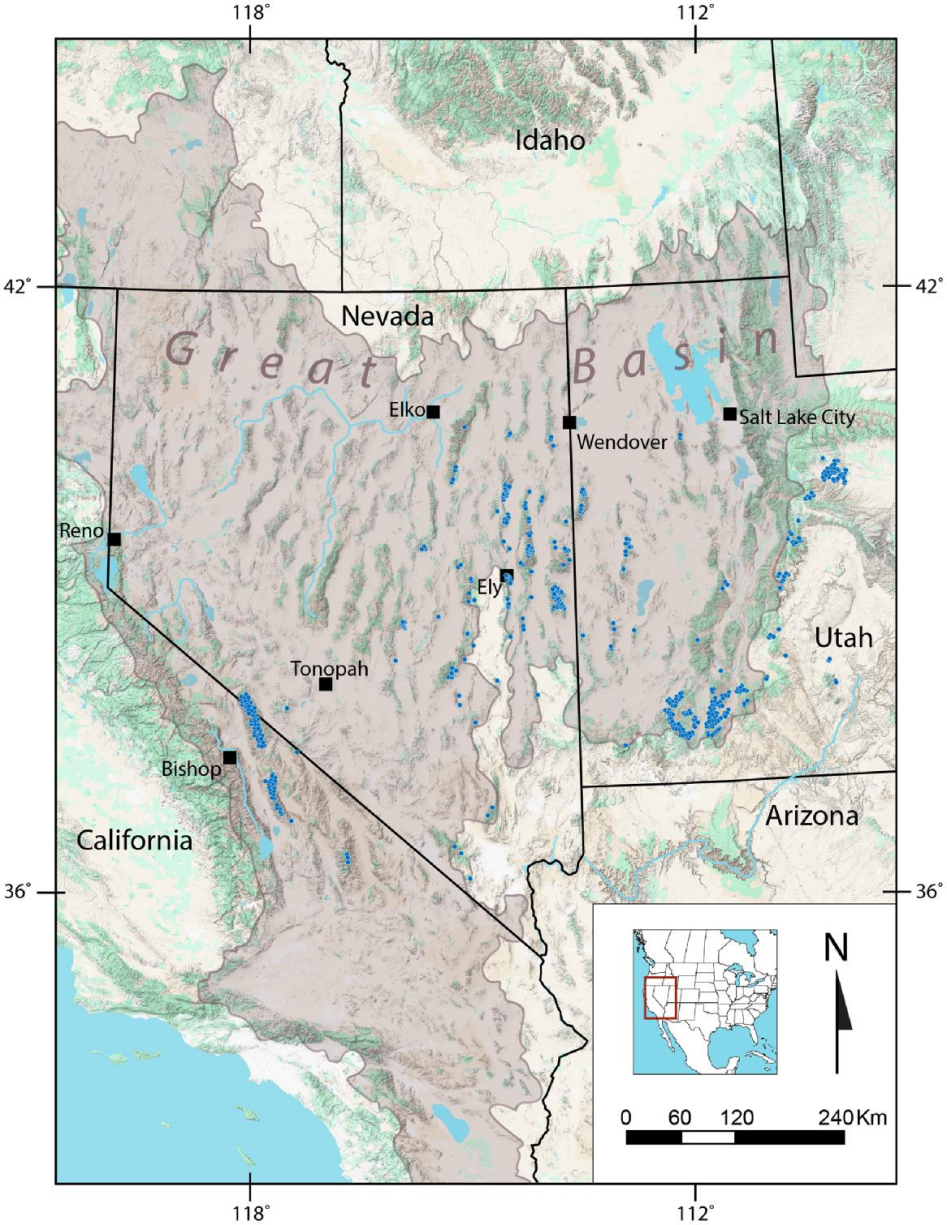
Current distribution of *Pinus longaeva* (small dots, as placed from site coordinates derived from Burchfield *et al*. 2021). The shaded, bounded area of the map denotes the hydrologic Great Basin, USA, encompassing almost entirely all the known *P. longaeva* distribution sites.

Great Basin bristlecone pine (*Pinus longaeva*) is a five-needle pine (subgenus *Strobus*, section *Parrya*, subsection *Balfouriana*, Pinaceae), endemic to high Great Basin mountains in eastern California, Nevada, and Utah, USA (Fig. 1). An ancient lineage, *P. longaeva* is most closely related to foxtail pine (*P. balfouriana*) of California and to Rocky Mountain bristlecone pine (*P. aristata*) of Colorado, New Mexico, and Arizona (Eckert and Hall 2006). *Pinus longaeva* currently grows in isolated patches on 43 mountain ranges of the Great Basin (Fig. 1; Burchfield *et al*. 2021), ranging from 2040 to ≥3600 m, with most populations growing above 2900 m where they form upper treeline (Hiebert and Hamrick 1984).

The continental climate of the Great Basin is cold and dry, with meager precipitation falling in winter, and arid, cool summers, accentuated at higher elevations (Grayson 2011). Although the Great Basin climate has common elements across the region, there are important differences, with summer precipitation increasing to the east and south, and winter precipitation increasing to the west. This suggests that the fundamental niche of *P. longaeva* likely varies across space and among genetically different subpopulations (Burchfield *et al*. 2021). *P. longaeva* grows on diverse substrates but thrives on soils of carbonate origin (dolostone, limestone; LaMarche 1969), which are high in phosphorus and low in the plant nutrients magnesium and calcium. The chemical attributes and rocky nature of Great Basin carbonate substrates inhibit growth of other conifers and woody shrubs, essentially eliminating competition for *P. longaeva*.

Harsh environmental conditions, including extreme temperature and precipitation, desiccating winds, severe substrate, low soil-water retention, and high soil-erosion potential contribute to conditions wherein *P. longaeva* often occurs as solitary, short-statured, wind-sculpted, and widely separated individuals (LaMarche 1969; Lanner 2002). At lower elevations and on more mesic sites, trees can grow straight and tall, with closed canopies, in dense forest stands.

Unusual as the stature, habitat, climate, and paleoecology of *P. longaeva* are, the species may be best known for its great longevity. With many trees living more than 4000 years (Schulman 1958), and the eldest recorded individual pushing or surpassing 5000 years, the species holds the record for greatest longevity of single individuals (ie genetic ramets versus clonal species). The oldest trees grow in stressful environments, such as the Methuselah tree, which is 4857-year old in 2025 (extending Schulman's 1958 age) and grows at the warm-dry lower treeline in the White Mountains of California. Another old tree, dubbed Prometheus, in the Snake Range of Nevada, was over 4800 years old when it was cut down in 1964 (Salzer and Baisan 2013). An unconfirmed tree in the White Mountain was provisionally dated in 2012 as 5065 years old (NPS 2021). A long-term goal of the *P. longaeva* genome project is to understand the genetic components to longevity in *P. longaeva*. Development of an annotated reference genome sequence is a first step on that discovery pathway.

## Methods and materials

### Reference tree

An approximately 2500-year-old Great Basin bristlecone pine tree (Burchfield *et al*. 2023) was selected from the Inyo National Forest near Bishop, California. The exact location of the tree is held in confidence to maintain its security. Cones and needle tissue were collected from this reference tree in 2022.

### DNA isolation and sequencing

#### Haploid seed megagametophyte

The protocol used to isolate the haploid megagametophyte tissue from a single fertilized *P. longaeva* seed was similar to that carried out for previous conifer genome sequencing projects (Zimin *et al*. 2014, Neale *et al*. 2024). Haploid genomic DNA was extracted from a single megagametophyte with the Omega Biotek E.Z.N.A.®SP Plant DNA Kit (D5511). The extraction followed the manufacturer's protocol with the following modifications: polyvinylpyrrolidone (0.01 g) was added to the tissue prior to lysis, and the lysis time was extended to 1.5 h. The extracted DNA was quantified on a Qubit 3.0 (5.02 ng/μl), a Nanodrop 2000C (A260/280: 1.56; A260/230: 2.01), and quality was evaluated on a TapeStation 4200 High Sensitivity D5000 ScreenTape device (fragment sizes >20,000 bp).

DNA from the megagametophyte was sequenced at the Johns Hopkins Genetic Resources Core Facility High Throughput Sequencing Center. First, DNA libraries were made using the Illumina DNA Prep Kit suitable for whole genome shotgun sequencing. Then sequencing was conducted on one lane of a NovaSeq 6000 SP flow cell, two lanes of a NovaSeq 6000 S1 flow cell, and four lanes of a NovaSeq 6000 S4 flow cell with Illumina 150 bp paired-ends sequencing with an approximate insertion size of 400 bp and non-overlapping ends, yielding approximately 140X coverage in short reads. The Illumina sequencing for this project generated a total of ∼3.4 trillion (3.4 × 10^12^) nucleotides in ∼ 22.7 billion reads.

#### Diploid needle tissue

A protocol similar to that used in previous conifer genome sequencing projects was used for Oxford Nanopore long-read sequencing (Scott *et al*. 2020; Neale *et al*. 2024). Sequencing with the Oxford Nanopore Technologies (ONT) platform precludes the use of DNA amplification with PCR because PCR introduces a bias towards shorter reads. This also then requires that we need more DNA per run because we cannot amplify the molecules we have, requiring more tissue to be extracted. A single seed does not provide enough DNA, DNA from needle tissue is thus used. High molecular weight DNA was extracted following the protocol described in Workman *et al*. (2018), which was customized for conifer needles. Briefly, tissue was ground in liquid nitrogen with a mortar and pestle for 10 min to properly disrupt tissue. This was followed by lysis in a nuclear isolation buffer (NIB) containing spermine, spermidine, triton, and β-mercaptoethanol in a 50 mL Falcon tube, rotating at 4 °C for 15 min. The resulting lysed sample was filtered through a 50 mL steriflip vacuum-driven filtration system with a 20 µm nylon net filter, then centrifuged 1900 × g for 20 min at 4 °C. The supernatant was decanted, and the pellet resuspended in 1 mL of NIB with a paintbrush. The resuspension was brought to a total volume of 15 mL NIB and centrifuged at 1900×g for 10 min at 4 °C. These steps were repeated (discard supernatant, resuspend pellet, and wash) until the supernatant was clear, usually 2 to 3 times. The final pellet was resuspended into 1 mL 1X HB buffer per gram of initial tissue. Nuclei were then spun at 7000 × g for 5 min at 4 °C, supernatant removed, and pellets snap frozen in liquid nitrogen and stored at −80 °C for later DNA extraction.

Extracted nuclei were then lysed and gDNA precipitated using the Circulomics Nanobind Plant Nuclei Big DNA kit (now PacBio Nanobind PanDNA kit 103-260-000). DNA was sheared to ∼50 kb with the Diagenode Megaruptor 3. The PacBio SRE XS kit (102-208-200) was used to remove fragments <5 kb and library preparation was performed according to the ligation sequencing kit V14 (LSK114, ONT). Then 1 μg of purified genomic DNA was input into the ligation sequencing kit V14 (LSK114, ONT). Samples were sequenced on R10.4 flowcells on the PromethION and then base-called using guppy 6.2.11, 6.3.9, or 6.4.6 depending on time of sequencing.

The same protocol was used to isolate plant nuclei as mentioned above (Workman *et al*. 2018) for Oxford Nanopore ultra-long read sequencing. Extracted nuclei were then lysed and gDNA precipitated using the NEB Monarch HMW DNA Extraction Kit for Tissue (T3060S). The Extraction EB (EEB) from the ultra-long DNA sequencing kit V14 (SQK-ULK114, ONT) was used to elute DNA. Then 750 μL of extracted uHMW gDNA was input into the ultra-long DNA sequencing kit V14 and library preparation was performed according to the published protocol (SQK-ULK114, ONT). Samples were sequenced on R10.4 flowcells on the PromethION and then base-called using guppy 6.5.7.

### Genome assembly

#### Nuclear genome

**Fig. 2.**
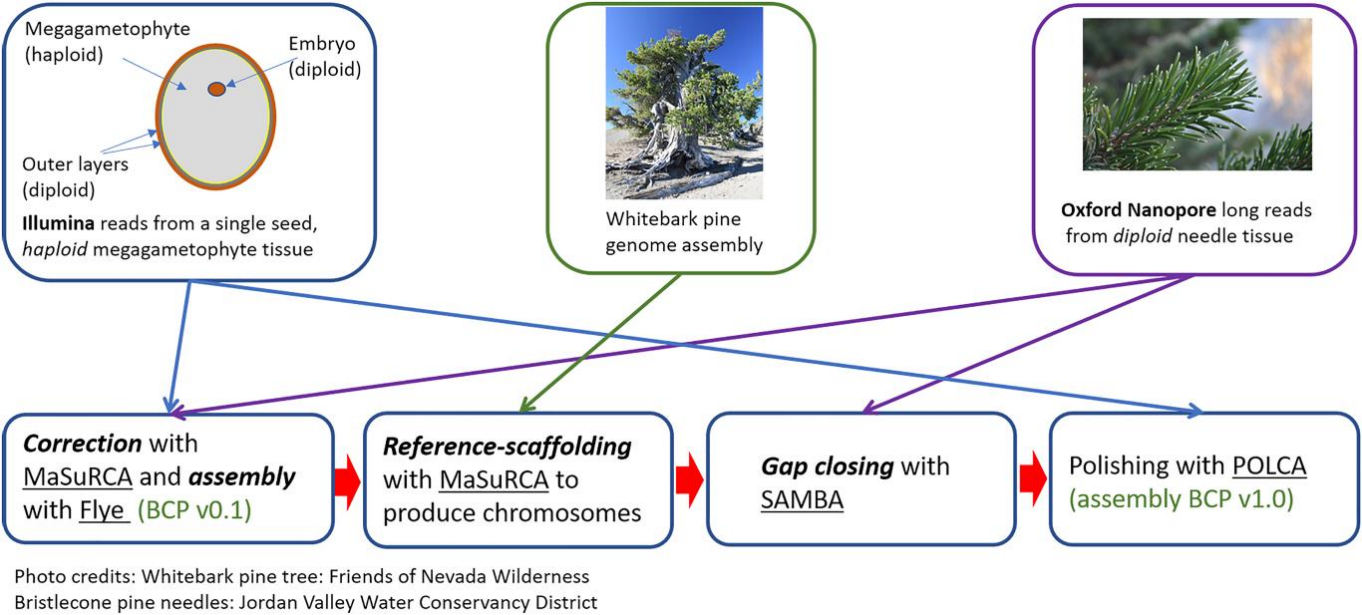
Overview of the sequencing and assembly strategy for the bristlecone pine genome.

The initial contig assembly used a hybrid approach combining ONT long reads and Illumina short reads (Fig. 2, Supplementary File 1). The use of haploid tissue, as our group pioneered in previous efforts to assemble conifer genomes (Neale *et al*. 2014), was designed to reduce genome complexity and improve assembly accuracy. Assembly was performed using MaSuRCA (v4.1.1), which began by transforming error-corrected Illumina reads into “super-reads,” which are longer sequences generated by extending reads uniquely based on k-mers in the Illumina reads (*k* = 99 for this assembly) as far as possible in the 5′ and 3′ directions. This step compressed 22.7 billion 150-bp reads (∼3.4 trillion bases) down to 123 million super-reads (∼51.6 billion bases) with an average length of 954 bp (Table 1).

**Table 1.** Statistics for intermediate assembly products: super-reads and mega-reads.

	Total sequence (bp)	Count	Average read size (bp)	N50 size (bp)
Super-reads	51,586,268,038	123,110,054	419	954
Mega-reads	413,169,936,111	27,951,917	14,781	23,422

N50 size is the size for which 50% of the total sequence is covered by reads above the N50 value.

Super-reads were then used to error-correct the ONT reads, producing “mega-reads,” which are highly accurate mini-assemblies of super-reads joined by exact overlaps of at least *k* = 99 bases guided by ONT reads. Note that each super-read can be used to correct many different ONT reads. At the end of this process, each ONT read was converted into one or more non-overlapping mega-reads. Since super-reads were derived from haploid Illumina data, ONT reads that represented highly divergent regions of the other haplotype were fractured into many small mega-reads and later eliminated from the assembly inputs. About 28 million mega-reads were generated, with an N50 length of 23,422 bp (Table 1).

The mega-reads were then assembled using a modified version of Flye (Kolmogorov *et al*. 2019) that was configured to bypass the initial consensus step, which is unnecessary when the input reads have a very low error rate. Bypassing this step dramatically reduced the overall computational load. After skipping consensus, Flye assembly continued with repeat resolution and scaffolding.

The resulting contigs (assembly v0.1, see Table 2) were scaffolded using two iterations of SAMBA (Zimin and Salzberg 2022), which joined contigs using long ONT reads. All ONT reads were used for this step. SAMBA was set to use 9 kb or longer matches (overlaps) in the first iteration and 4 kb in the second. Gaps were filled using the consensus computed from multiple ONT reads. This yielded assembly version 0.2.

**Table 2.** Statistics for intermediate genome assembly steps, from contig assembly to final polishing and scaffolding.

Assembly version	Total sequence (bp)	Scaffold N50 (bp)	Contig N50 (bp)	Number of scaffolds	Number of contigs	Sequence accuracy %
Initial contig assembly, v0.1	23,803,308,277	604,758	600,883	136,408	136,834	99.99
After SAMBA scaffolding, v0.2	23,828,892,812	625,299	621,306	130,610	131,036	99.99
After reference-based scaffolding, v0.3	23,823,713,014	1,200,043,644	621,355	80,998	123,299	99.99
After additional gap closing	23,824,718,022	1,195,948,424	626,009	80,998	122,880	99.99
After polishing	23,824,205,140	1,195,922,217	625,997	80,998	122,880	99.998

Next the assembly was re-scaffolded with a reference-based scaffolding approach, using the recently published assembly of a close relative, whitebark pine (*Pinus albicaulis*) (Neale *et al*. 2024) as the reference. The *P. albicaulis* assembly is highly contiguous, with most chromosomes assembled into a single scaffold each. The reference-based scaffolding (included in a module in MaSuRCA) took the assembly version 0.2 contigs and arranged them into larger scaffolds using their alignments to *P. albicaulis*. During this step, 7737 short contigs (average length 669 bp) containing 5,179,708 bp of sequence were eliminated because they aligned completely to the interior of larger contigs. This yielded assembly version 0.3.

Additional gap closing was performed with SAMBA, using ONT reads again and restricting the scaffolder to allow only merges of contigs that were immediately adjacent in a scaffold. This process closed 419 gaps, increasing the N50 contig size to 626,009 bp. The assembly was polished using POLCA (Zimin and Salzberg 2020), improving consensus quality from ∼1 error per 10 Kb to ∼2 errors per 100 Kb).

#### Chloroplast genome

As expected, the chloroplast and mitochondria genomes were present in relatively high concentrations compared to the primary chromosomes in all sequencing libraries. To assemble the chloroplast genome, all ONT ultralong reads were first aligned with Minimap2 (Li 2018) to the published chloroplast genome of loblolly pine (*P. taeda*) (Zimin *et al*. 2014), (NCBI accession NC_021440.1) (Supplementary File 1). Reads longer than 20 Kb that had alignments spanning at least 75% of the read were retained, which yielded 576 reads containing 15,063,428 bp of sequence. These reads were assembled with Flye to produce a single circular contig containing 119,863 bp. To polish the assembly, Illumina reads from a single-sequencing library were mapped to this contig, and reads that aligned over the entire span of the read with >90% identity were extracted. The chloroplast contig was then polished with the Illumina reads, which yielded a perfect assembly with no detectable errors; ie every base was supported by multiple individual reads.

#### Mitochondrial genome

In order to filter mitochondrial DNA from nuclear and chloroplast DNA, Minimap2 (Li 2018) was used to map *P. longaeva* ONT ultralong reads (99.3 Gbp) to the mitochondrial genomes of closely related conifers, including the sugar pine (*P. lambertiana*) (Stevens *et al*. 2016) and Siberian larch (*Larix sibirica*) (Putintseva *et al*. 2020) (Supplementary File 1). The extracted reads were assembled using Flye in metagenome mode, which yielded 25,211 contigs containing 1.13 Gbp of sequence. To refine this set of contigs, three filtering steps were employed. First, mitochondrial protein sequences from *L. sibirica* were mapped to the candidate contigs using Minimap2, yielding 92 alignments to 27 candidate contigs containing ∼4 Mbp of sequence. Second, short reads from a single library were aligned to the candidate contigs. The 27 high confidence contigs from the previous step all had high Illumina coverage, exceeding 120X, which provided evidence that the high confidence contig set was reliable. Given that the average Illumina coverage from the selected library was approximately 8X, we identified other contigs with at least 120X coverage to add to the mitochondrial contig set. Finally, the candidate contigs were aligned to the *L. sibirica* mitogenome, and contigs from the candidate set that aligned over 50% of their length were retained in the high confidence set.

These high confidence contigs were then aligned to the *P. longaeva* nuclear genome. Contigs that aligned over at least 90% of their length to any nuclear sequence were excluded, as they likely belonged to the nuclear genome. None of the 27 contigs with protein alignments ended up in the excluded set. The filtered set of mitochondrial candidates contained 79 contigs with a total length of 8.67 Mbp. We then scaffolded the mitochondrial contigs using ONT ultra-long reads and SAMBA, which yielded a single scaffold containing 75 contigs and a total length of 8.68 Mbp.

Following scaffolding, we polished the preliminary assembly with POLCA, using reads from a single Illumina library (∼5 billion 150-bp reads). This increased the estimated assembly accuracy from 99.48% to 99.996%, which corresponds to ∼1 error every 25,000 bases.

### Gene annotation

#### Protein coding gene and long noncoding RNA annotation

Genome annotation was performed using EviAnn v2.0.3 (Zimin *et al*. 2026), which combines transcriptional and protein sequence evidence to produce high-confidence gene models without reliance on *ab initio* gene prediction. Functional annotation was performed using a BLASTP (Camacho *et al*. 2009) search of all annotated protein-coding genes against the UniProt-SwissProt database (Bairoch *et al*. 2005). For each gene, the top-scoring match was used to assign a putative gene name, and the label “Similar to” was added to the name to indicate homology-based functional inference. Organelle (mitochondria and chloroplast) annotations were produced along with the nuclear genome annotations by using the “–mito_contigs” switch in EviAnn and supplying a list of contigs names containing mitochondrial and chloroplast sequences. This enabled EviAnn to use a different set of stop codons (TAA and TAG only) for the organelle genomes. Transfer RNAs (tRNAs) were annotated using tRNAscan-SE version 2.0.5. BUSCO version 5.8.3 was used to estimate completeness of the assembly and annotation, using the “embryophyte_odb10” database of plant orthologous proteins, which contained 1614 single-copy plant proteins (Simão *et al*. 2015; Manni *et al*. 2021).

#### Transcriptomic evidence

EviAnn uses RNA-seq data and proteins from related species as evidence to produce annotation. The RNA-seq data do not have to be from the same species, as long as they are close enough to permit alignment of RNA-seq reads to the assembly. We used 16 RNA-seq datasets from NCBI BioProject PRJNA703422 (Jin *et al*. 2021) as transcriptional evidence for the annotation. Only two of RNA-seq datasets were from *P. longaeva*, and the rest were from close relatives. Table 3 lists the RNA-seq data used for annotation, including NCBI SRA accession numbers, read counts, and species names.

**Table 3.** Description of RNA-seq data used for gene annotation.

SRA identifier	Species	Total bases	Location of sample collection
SRR13823435	*Pinus aristata*	5,928,113,736	USA: San Isabel National Forest
SRR13823439	*P. rzedowskii*	9,742,220,800	Mexico
SRR13823502	*P. nelsonii*	11,419,731,400	United Kingdom: Royal Botanic Garden Edinburgh
SRR13823511	*P. monophylla*	5,357,668,624	USA: California
SRR13823586	*P. edulis*	7,607,857,824	USA: Northern New Mexico
SRR13823588	*P. edulis*	3,847,284,728	USA: Northern New Mexico
SRR13823620	*P. aristata*	6,910,733,280	unknown
SRR13823621	*P. cembroides*	8,340,949,296	USA: Texas
SRR13823622	*P. cembroides*	5,501,002,680	USA: Texas
SRR13823449	*P. remota*	10,482,400,000	Mexico
SRR13823452	*P. quadrifolia*	6,996,455,200	Canada: UBC Botanical Garden
SRR13823519	*P. monophylla*	6,960,680,226	USA: Tulare, California
SRR13823529	*P. balfouriana*	6,935,823,120	USA: Inyo, California
SRR13823538	*P. longaeva*	7,123,173,120	USA: California
SRR13823539	*P. longaeva*	4,303,053,470	USA: California
SRR13823540	*P. balfouriana*	4,212,198,936	USA: Inyo, California

In addition to the RNA-seq reads, EviAnn used 825,777 protein sequences from 827 plant species that belong to classes Pinopsida and Lycopodiopsida and four species of the angiosperm genus *Populus* as evidence for both gene structure and function. The protein sequences were obtained from NCBI GenBank by going to “Taxonomy” and then downloading all protein sequences for all species belonging to those two classes and the four *Populus* species. The entire list of 831 species is provided as Supplementary Table 1.

## Results and discussion

This paper reports the sequencing, assembly, and annotation for *P. longaeva*. This is the seventh conifer genome sequence completed by this collaborative team in the past 15 years. The large increase in sequence contiguity over time is clear, due to advances in sequence read technology and assembly algorithms (Zimin and Salzberg 2022). All seven reference genomes we have reported, with the exception of *P. longaeva*, include a deep transcriptome resource. Although we did not generate a *P. longaeva* transcriptome, we were able to use transcriptomes from related species to aid annotation. Development of a deep transcriptome resource specifically for *P. longaeva* is a high priority to support future research in *P. longaeva* genomics research.

### K-mer-based estimates of the genome size

The genome size was estimated by counting k-mers (*k* = 27) in a subset of Illumina reads with Jellyfish (Marçais and Kingsford 2011). Jellyfish outputs a histogram of k-mer count *C* and the number of k-mers with count *C* in the Illumina reads (see Supplementary Fig. 1 in Supplementary File 1). The k-mer coverage was estimated to be 49 from the mode of the histogram and the minimum count of a non-error k-mer was found to be 19 from the first trough in the histogram. Two approaches to estimating the genome size were implemented. The first approach was purely k-mer based. The total number of non-error k-mers in the genome (count *C* in the Illumina reads > 19) was computed and then divided by the k-mer coverage = 49. This computation yielded an estimate of 21.6 Gbp for haploid genome size. Typically, this way of estimating the genome size provides a lower-bound estimate. Another way to estimate the genome size is to estimate the read coverage as k-mer coverage times the ratio of the average read length (150) to the number of 27-mers in each read (150-27 + 1). Then the genome size can be estimated as a ratio of the total sequence in the Illumina reads to the read coverage. This way of estimating the genome size typically provides an upper bound for the genome size. The total amount of sequence in the subset of reads used for this evaluation was 1639 Gbp, the estimated read coverage was 59.3 and this estimate yielded a 27.7 Gbp haploid genome size. Thus, k-mer-based methods yield a range of 21.6 to 27.7 Gbp for the actual genome size, consistent with our assembly size of 23.8 Gbp (see Table 2).

Using the same subset of Illumina reads and another k-mer-based tool Merqury (Rhie *et al*. 2020), we have estimated genome completeness at 94.9% and an average base quality of Q41 translating to less than 1 error per 12,500 bp. This estimate is an upper bound of the number of errors, because Illumina data were collected from a single haplotype, but the assembly included ONT reads from both haplotypes, and thus divergent sequences from the alternative haplotype were counted as “errors”. A more reliable estimate of the sequence quality was computed by aligning the Illumina reads to the assembly and counting as errors bases in the assembly where all Illumina reads disagreed with the assembly. This estimate (about 1 error per 200,000 bp or 99.998%) is shown in Table 2.

### Genome assembly results

Assembly of the *P. longaeva* genome was performed using a hybrid strategy, similar to the one used in our previous large conifer genome projects (Zimin *et al*. 2017; Scott *et al*. 2020; Neale *et al*. 2022, 2024) and outlined in Fig. 2. Haploid Illumina data from a single megagametophyte were used to produce an estimated 140X genome coverage in paired 150-bp Illumina reads (Table 4). In addition, needle tissue was used to produce ∼13X genome coverage in long ONT reads and another ∼3X coverage in “ultra-long” ONT reads, for a total of ∼16X long-read coverage. After polishing (see Methods), the final assembly had an estimated overall sequence error rate of less than 1 error per 200,000 bp, which is a slight improvement over the error rate in the *P. albicaulis* genome assembly produced following the same strategy (Neale *et al*. 2024). One unusual genomic feature of *P. longaeva* is the very large size of its mitochondrial genome (mitogenome), which is much larger than typical mitogenomes but roughly similar to those of other conifers. Overall statistics for the nuclear and organelle genome assemblies are shown in Table 5.

**Table 4.** Sequencing data used for the *P. longaeva* genome assembly.

Sequencing technology	Total sequence (bp)	Read count	N50 size (bp)
Illumina	3,398,589,869,700	22,657,265,798	150
Oxford nanopore (ONT)	406,216,997,203	24,471,387	24,315
ONT ultra-long reads	99,262,889,529	3,927,667	49,190

**Table 5.** Contig and scaffold size and quality statistics for the *P. longaeva* genome assembly.

Assembly	Total sequence (bp)	Scaffold N50 (bp)	Contig N50 (bp)	Number of scaffolds	Number of contigs	Contig consensus quality %
Primary assembly v1.0	23,824,205,140	1,195,922,217	625,997	80,998	122,880	99.998
Mitochondrial genome v1.0	8,639,613	196,416	195,473	75	76	99.999
Chloroplast genome v1.0	119,863	119,863	119,863	1	1	100

Contig consensus quality is the percentage of error-free bases in the assembly. N50 size is the value *N* for which 50% of the assembly is contained in scaffolds/contigs of size *N* or larger.

### Genome annotation

We annotated 21,364 protein coding genes that contained 62,959 protein-coding transcripts; ie about three transcripts per gene (Table 6). Because two or more transcripts might have the same coding sequence and differ only in the UTR regions, the 62,959 protein-coding transcripts contain only 44,721 distinct protein sequences in the nuclear genome. It was possible to assign biological function to 86% of the protein-coding transcripts by alignment of protein sequences to the UniProt-Swissprot database of curated functional proteins (Bairoch *et al*. 2005). Chloroplast and mitochondrial gene annotation statistics are shown separately in Table 6.

**Table 6.** Annotation statistics for protein-coding genes, lncRNAs, tRNAs, and pseudogenes.

Genome	Genes				Transcripts			
	Protein coding	lnc RNA	Pseudo-genes	tRNA	Protein coding	lnc RNA	Function assigned	Distinct proteins
Nuclear	21,364	8,349	385	15,300	62,959	8,316	54,075	44,721
Chloroplast	15	1	1	30	17	0	15	15
Mitochondrial	175	14	8	136	191	7	170	136

Pseudogenes include only processed pseudogenes. “Function assigned” indicates genes for which a named ortholog could be found in the UniProt database. “Distinct proteins” indicates the number of all distinct protein sequences in the annotations. This number is smaller than the number of protein-coding transcripts.

The BUSCO software was run to estimate annotation completeness, although it is based on a database of single-copy proteins from angiosperms rather than gymnosperms (see Materials and Methods). The assembly contained 76.5% of the BUSCO single-copy proteins as complete sequences, of which 68.1% were in a single copy and 8.4% were duplicated. 14.6% of the remaining BUSCO proteins were fragmented and 8.9% were missing.

### Insights into longevity in *P. longaeva*

Understanding the environmental and genetic basis, and the complex interaction between environment and genetics, of longevity in the oldest living organisms on earth is a topic of fundamental interest in biology. Piovesan and Biondi (2021) published a comprehensive review on tree longevity that focuses on environmental factors and tree phenotypes and also includes discussion of genetic factors. Many possible environmental and tree phenotype factors have been identified that may determine longevity specifically in *P. longaeva* (Lanner and Connor 2001; Lanner 2002; Lanner 2007). Obvious environmental factors are the extreme cold and dry environment, carbonate substrates, and rarity of fire where *P. longaeva* is found. Such environments would reduce the number of competitors and pathogens. Lanner (2007) argues, however, that two of *P. longaeva's* neighbors at just slightly lower elevation, *P. flexilis* and *P. albicaulis,* do not live nearly as long, so there must be something genetically inherent to *P. longaeva*.

Lanner and Connor (2001) conducted empirical studies with three phenotypes in *P. longaeva* to argue that it does not senesce. They showed that (i) tracheid diameter does not decrease with age, (ii) needle number in winter buds and spring shoot growth does not decrease with age, and (iii) pollen viability, seed weight, seed germinability, and seedling biomass accumulation do not decline with age, thus inferring that there is no accumulation of deleterious mutations. These results would suggest that gene regulatory processes controlling these phenotypes do not significantly change with aging. Lanner (2002) also points to the sectored vascular system in *P. longaeva* that allows damaged stems to maintain water and nutrient transport in the stem. All these factors are phenotypes, but the inference is that they are genetically determined and not environmentally determined.

Several other phenotypes may indirectly support longevity in *P. longaeva*. These include sectoral growth (“strip bark”); dense, resin-rich wood that inhibits wood-decomposing fungi (stems do not rot over millennia); needle persistence to 50 years; and resistance to bark beetles (inhibited by terpenoid composition, Bentz *et al*. 2017). The latter two traits are unique among pines to *P. longaeva*, and in combination with the rare former two, enable trees to grow to a great age without dying. These traits likely have genetic bases.

A very comprehensive review on the genetic and epigenetic mechanisms of longevity in trees did not include genomic data from *P. longaeva*, simply because such data did not exist in 2023 (Batalova and Krutovsky 2023). Nevertheless, the observations, inferences, and conclusions they made from genomic and transcriptomic data from a number of trees, including one species of *Pinus*, serves as a long list of potential genetic mechanisms for longevity in *P. longaeva* once transcriptomic data become available. Just a few of the genomic and transcriptomic observations they made include numbers and enhanced expression of disease resistance, defense, DNA repair, leaf meristem, wound healing, and callus formation genes.

Nucleotide-binding, leucine-rich repeat receptor (NLR) genes play a central role in plant immunity by detecting pathogen attacks and triggering defense responses. They encode intracellular receptors that recognize specific pathogen-derived molecules and initiate immune signaling cascades. NLR proteins exhibit a modular structure, typically comprising an N-terminal coiled-coil (CC) domain, a Toll/interleukin-1 receptor (TIR) domain, or less commonly, an RPW8-like CC domain. These are followed by a conserved nucleotide-binding domain (NB-ARC) and a C-terminal region containing a variable number of leucine-rich repeats (LRRs) (Monteiro and Nishimura 2018).

NLR-Annotator pipeline version 2.1 (Steuernagel *et al*. 2020) was used in this study to identify NLR genes in the *P. longaeva* genome. The resulting annotations were classified as “complete” if all three canonical domains (CC/TIR, NB-ARC, LRR), were present, or “partial’ if one of the domains was missing. The same pipeline was also applied to the genomes of other long-lived conifers such as *Sequoiadendron giganteum* (GenBank accession GCA_007115665.2), *Sequoia sempervirens* (GenBank accession GCA_007258455.2), and *P. albicaulis* (GenBank accession GCA_034641835.3) and to the genome of the rather short-lived conifer *Pinus taeda* (GenBank accession GCA_000404065.3). The NLR gene counts were normalized by ploidy of the assemblies (three for *S. sempervirens* and one for the others). Table 7 lists counts of complete and partial NLR genes found in the assemblies by the NLR-annotator. While Batalova and Krutovsky (2023) suggested the numbers and enhanced expression of disease resistance genes are positively correlated with longevity, it seems clear that the counts of complete and partial NLR genes are not a component of that correlated set of genes. They are not positively correlated with longevity, at least for the species in Table 7.

**Table 7.** Estimated NLR gene counts for *Pinus longaeva*, *P. albicaulis*, *P. taeda*, *Sequoiadendron giganteum*, and *Sequoia sempervirens*.

Species	Estimated tree Age (years)^a^	Complete NLR genes	Partial NLR genes
*Pinus longaeva*	∼2500	403	1148
*Pinus albicaulis*	∼150	599	1748
*Pinus taeda*	8	954	2424
*Sequoiadendron giganteum*	∼1360	410	565
*Sequoia sempervirens*	∼1390	690	1002

^a^Sources for sampled tree age: *P. longaeva*—this paper; *P. albicaulis*—Neale *et al*. 2024; *P. taeda*—F. Isik, personal communication, 23 February 2026; S*equoia giganteum*—Scott *et al*. 2020; *Sequoia sempervirens*—Neale *et al*. 2022.

Flanary and Kletetschka (2005) measured telomere length in *P. longaeva* from trees ranging in age from 20 to 3500 years and observed both an increase in telomere length and telomerase activity with age. The telomeric sequences for plants and human are well known, they are CCCTAAA for plants and CCCTAA for the human genome. To estimate the telomere lengths in a few conifers, 10 million Illumina reads were examined for *P. taeda*, *P. longaeva*, *P. albicaulis, S. giganteum*, and *S. sempervirens*. Illumina reads used to measure conifer telomere lengths were sequenced from megagametophyte tissue for all genomes. The human genome was included for validation of the method, as it is well known that an adult human has telomere lengths of 5 to 8 Kbp, with a loss of 20 to 40 bp per year of life. Illumina reads were trimmed to the first 100 bp and then reads containing five-copy telomere sequences were counted. The average telomere lengths were then estimated as $L=G\frac{T}{2RC}$, where *G* is the genome size, *T* is the number or reads that contained telomeric sequences, *R* is the total number of reads examined (10 million), and *C* is the number of chromosomes. Table 8 shows the comparison between the conifers and the human genome. The method yielded the result for human genome that matched the estimates. *P. longaeva* has the second longest telomeres of all species that were examined, longer than *S. giganteum*, *S. sempervirens*, and *P. albicaulis*, but shorter than loblolly pine (*P. taeda*).

**Table 8.** Average telomere lengths estimated from the Illumina reads for *Pinus taeda*, *P. longaeva*, *Sequioadendron giganteum*, *Sequoia sempervirens*, *P. albicaulis*, and human (10 million Illumina reads examined for each species).

Species	Estimated tree age (years)	Number of telomeric reads	Genome size (Gbp)	Number of chromosomes	Average telomere length (bp)
*Pinus taeda*	8	319	24	12	31,900
*Pinus longaeva*	∼2500	155	23.8	12	15,371
*Sequoiadendron giganteum*	∼1360	318	8.9	11	13,009
*Sequoia sempervirens*	∼1390	275	26.4	33	11,000
*Pinus albicaulis*	∼150	43	27.6	12	4945
Human		811	3	23	5289

Sources of tree ages as in Table 7.

We would have liked to estimate average telomere length in all species across several time points in their lifetime, similar to what Flanary and Kletetschka (2005) did for *P. longaeva*; however, sequence data do not exist for these species to produce estimates across this timeframe. Thus, we obtained a single estimate for each species from the DNA sequence available from the one reference tree we have previously sequenced. While the ages of these sequenced trees differ greatly, a general trend emerges that all the long-lived tree species have much greater estimates of average telomere length than human, with the outstanding exception of the shorter-lived *P. taeda* which has the highest estimate of all (Table 8). Might the average telomere length estimate from the short-lived loblolly pine be an outlier? A deeper understanding of the relationship between telomere length and longevity in conifers will come when many more conifers of all ages have been sequenced and average telomere lengths estimated.

## Conclusion

While *P. longaeva* is a conifer species of no commercial value, it is one of significant ecological and evolutionary interest. The reference sequence will serve as an important resource as future investigators seek to identify more genetic mechanisms underlying longevity in *P. longaeva.* Additionally, the reference sequence will also serve as an important genetic resource as researchers seek to discover genes determining adaptation to the extreme environments where these trees are found and attempt to infer if *P. longaeva* will remain adapted to these locations as climate changes.

## Supplementary Material

jkag064_Supplementary_Data

## Data Availability

The bristlecone pine read data and annotated assembly have been deposited in NCBI SRA and GenBank under BioProject PRJNA1332257 (BCP1.0 JBWCIA000000001). Details on assembly and annotation software and command line parameters used are provided in Supplementary File 1. Supplemental material available at G3 online.
