# Supplementary material for: A reference genome sequence for the exceptionally long-lived Great Basin bristlecone pine, Pinus longaeva
Source: G3 (Bethesda). 2026 Mar 17;16(6):jkag064. doi: 10.1093/g3journal/jkag064 (PMC13233319; doi:10.1093/g3journal/jkag064)
Supplement: jkag064_Supplementary_Data [file jkag064_supplementary_data.zip › Supplementary_File_1_G3-2026-406665.pdf]

Supplementary File 1
Bristlecone pine genome assembly and annotation process details

Supplemental information for:

Genome Report: A reference genome sequence for the exceptionally long-lived Great Basin bristlecone pine, *Pinus longaeva*; David B. Neale, Aleksey V. Zimin, Constance I. Millar, Patrick E. McGuire, Jessica A. Hosea, Edward Li, Daniela Puiu, Winston Timp, Steven L. Salzberg

K-mer based estimates of the genome size

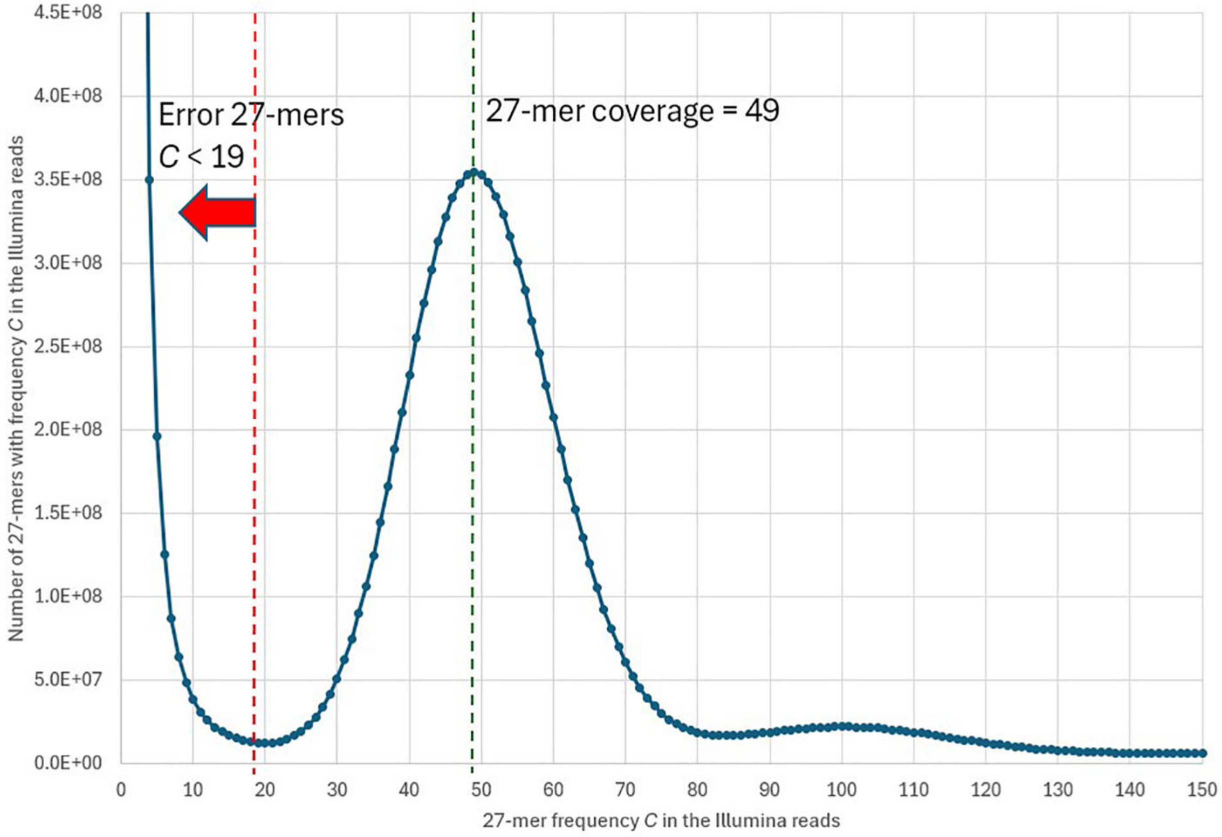

Figure S1. Histogram of k-mer (k=27) counts computed by counting the frequencies of 27-mers in a subset of Illumina reads sequenced from a single Great Basin bristlecone pine megagametophyte.

Assembly of nuclear genome

We performed initial contig assembly with MaSuRCA assembler version 4.1.1. We used all Illumina reads and Oxford Nanopore reads (ONT). All defaults were used on the command line. Below are the parameters from the MaSuRCA configuration file:

```
PARAMETERS
EXTEND_JUMP_READS=0
GRAPH_KMER_SIZE = 99
```

```
USE_LINKING_MATES = 0
USE_GRID=1
GRID_ENGINE=MANUAL
GRID_QUEUE=all.q
GRID_BATCH_SIZE=5000000000
LHE_COVERAGE=25
LIMIT_JUMP_COVERAGE = 300
CA_PARAMETERS = cgwErrorRate=0.15
CLOSE_GAPS=1
NUM_THREADS = 64
JF_SIZE = 200000000
SOAP_ASSEMBLY=0
FLYE_ASSEMBLY=1
END
```

The assembly process required a computer with 32 cores and 2TB of RAM. We ran individual mega-reads correction jobs on several computers with 1TB of AN and 24 to 32 cores. The output of the assembly was an assembly.fasta file. We followed the assembly with scaffolding with the SAMBA scaffolder, which used ONT reads to scaffold assembled contigs. SAMBA is distributed with MaSuRCA. We used the following command line with SAMBA:

```
samba.sh -r assembly.fasta -q ont_reads.fa -t 32 -m 5000 -o 2000
```

Following this step we reference-scaffolded the resulting assembly (assembly.fasta.scaffolds.fa) with MaSuRCA chromosome scaffolder using the Whitebark pine assembly version 1.0 as reference:

```
chromosome_scaffolder.sh -t 32 -r wbp1.0.fa -q assembly.fasta.scaffolds.fa -nb
```

This resulted in the reference-scaffolded assembly WBP_1.0.fa.assembly.fasta.scaffolds.fa.split.reconciled.fa. We then performed an additional round of gap closing with the script close_scaffold_gaps.sh, which is a wrapper for SAMBA scaffolder (also distributed with MaSuRCA):

```
close_scaffold_gaps.sh -t 32 -r WBP_1.0.fa.assembly.fasta.scaffolds.fa.split.reconciled.fa -g
ont_reads.fa -d ont -i 90
```

We renamed the output to assembly.refscaff.split.joined.fa. Finally, we polished the resulting assembly with POLCA polisher (also distributed with MaSuRCA suite) using a subset of Illumina reads:

```
polca.sh -t 32 -r ‘BCP_MEG_2_Elution_S1_L001_R1_001.fastq.gz
BCP_MEG_2_Elution_S1_L001_R2_001.fastq.gz BCP_MEG_2_Elution_S1_L002_R1_001.fastq.gz
BCP_MEG_2_Elution_S1_L002_R2_001.fastq.gz BCP_MEG_2_Elution_S1_L003_R1_001.fastq.gz
BCP_MEG_2_Elution_S1_L003_R2_001.fastq.gz BCP_MEG_2_Elution_S1_L004_R1_001.fastq.gz
```

```
BCP_MEG_2_Elution_S1_L004_R2_001.fastq.gz BCP_MEG_2_Elution_S1_L005_R1_001.fastq.gz
BCP_MEG_2_Elution_S1_L005_R2_001.fastq.gz BCP_MEG_2_Elution_S1_L006_R1_001.fastq.gz
BCP_MEG_2_Elution_S1_L006_R2_001.fastq.gz BCP_MEG_2_Elution_S1_L007_R1_001.fastq.gz
BCP_MEG_2_Elution_S1_L007_R2_001.fastq.gz BCP_MEG_2_Elution_S1_L008_R1_001.fastq.gz
BCP_MEG_2_Elution_S1_L008_R2_001.fastq.gz' -a assembly.refscaff.split.joined.fa
```

This resulted in assembly.refscaff.split.joined.fa.PolcaCorrected.fa, which was the final product of the assembly process. We then ran POLCA one more time to estimate the error rate in the assembly.

Assembly of mitochondrial and chloroplast genomes

Chloroplast. We assembled the chloroplast genome by first aligning ONT reads to the complete chloroplast genome of *Pinus taeda* (loblolly pine, NCBI RefSeq ID NC_021440.1) with minimap2:

```
minimap2 -t 32 -x map-ont NC_021440.1.fa ont_reads.fa >minimap.paf
```

We then used reads longer than 20 Kb that had alignments spanning at least 75% of the read, which yielded 576 reads containing 15,063,428 bp of sequence (ont_chloroplast.fa). These reads were assembled with Flye:

```
flye -t 32 -g 120000 –nano-raw ont_chloroplast.fa -g chloroplast
```

to produce a single circular contig containing 120,195 bp. We then aligned Illumina reads from a single library to this contig with minimap2:

```
minimap2 -t 32 chloroplast.fasta BCP_MEG_2_Elution_S1_L001_R1_001.fastq.gz | awk '{if($4-$3>$2*.9) print $1}' > illumina_chlor.readnames.txt
```

We extracted all Illumina reads whose alignment span was at least read_length*.9 (see above):

```
zcat BCP_MEG_2_Elution_S1_L001_R1_001.fastq.gz | fastqExtract.pl illumina_chlor.readnames.txt > Illumina_chlor_polishing.fastq
```

We then polished the choloplast assembly with POLCA using these reads:

```
polca.sh -t 32 -a chloroplast.fasta -r Illumina_chlor_polishing.fastq
```

which resulted in the polished chloroplast genome chloroplast.fasta.PolcaCorrected.fa

Mitochondrial genome. Minimap2 was used to map bristlecone pine ONT ultralong reads (99.3 Gbp) to the mitochondrial genomes of closely related conifers, including the sugar pine (*Pinus lambertiana*) and Siberian larch (*Larix sibirica*):

```
minimap2 -t 32 pita_slar_mito.fasta ont_ul.fa | awk ‘{if($4-$3>$2*.5) print $1}’ > ont_mito.readnames.txt
```

We extracted reads that aligned for over 50% of the length:

```
cat ont_ul.fastq |fastqExtract.pl ont_mito.readnames.txt > ont_ul_mito.fastq
```

The extracted reads were assembled using Flye in metagenome mode:

```
flye -g 10000000 -t 32 –meta –ont-raw ont_ul_mito.fastq -o mito
```

Then we aligned mitochondrial protein sequences from *Larix sibirica* to the candidate contigs using miniprot:

```
miniprot -t 32 mito.fasta larix_prot.faa |awk ‘{print $1}’ > mito_prot_keep.txt
```

This yielded 92 alignments to 27 candidate contigs containing ~4Mbp of sequence (contig names mito_prot_keep.txt).

We aligned short reads from a single library to the candidate contigs:

```
minimap2 -t 32 mito.fasta BCP_MEG_2_Elution_S1_L001_R1_001.fastq.gz | awk ‘{if($4-$3>$2*.9) print $0}’ > illumina_mito.paf
```

We identified contigs with at least 120X coverage to add to our mitochondrial contig set (mito_add_illumina.txt). Then, the candidate contigs were aligned to the Siberian larch mitogenome, and contigs from the candidate set that aligned over 50% of their length were retained in the high confidence set.

```
minimap2 -t 32 mito.fasta larix_genome.fasta | awk ‘{if($4-$3>$2*.5) print $1}’ > mito_add_larix.txt
```

Finally we extracted contigs that were in one of the three sets from the initial assembly:

```
ufasta extract -f <(cat mito_prot_keep.txt mito_add_illumina.txt mito_add_larix.txt) mito.fasta > mito.prelim.fa
```

We then aligned contigs in mito_prelim.fa file to the BCP_1.0 nuclear genome:

```
minimap2 -t 32 BCP_1.0.fa mito.prelim.fa| awk ‘{if($4-$3>$2*.9) print $1}’ > mito_exclude.txt
```

and excluded any contigs that mapped with over 90% span to any nuclear contig:

```
ufasta extract -v -f mito_exclude.txt mito.prelim.fa > mito_final.fa
```

This yielded the final mitochondrial assembly mito_final.fa. We then aligned Illumina reads from a single library to the mito contigs with minimap2:

```
minimap2 -t 32 mito_final.fa BCP_MEG_2_Elution_S1_L001_R1_001.fastq.gz | awk '{if($4-$3>$2*.9) print $1}' > illumina_mito.readnames.txt
```

We extracted all Illumina reads whose alignment span was at least read_length*.9 (see above):

```
zcat BCP_MEG_2_Elution_S1_L001_R1_001.fastq.gz | fastqExtract.pl illumina_mito.readnames.txt > Illumina_mito_polishing.fastq
```

We then polished the mito assembly with POLCA using these reads:

```
polca.sh -t 32 -a mito_final.fa -r Illumina_mito_polishing.fastq
```

which resulted in polished mitochondrial genome mito_final.fa.PolcaCorrected.fa. We repeated the polishing process 3 times, using the same set of reads, until the resulting number of errors in the mito_final.fa.PolcaCorrected.report file, produced by POLCA, converged to a small number.

Annotation

We then annotated the complete assembled genome consisting of the nuclear, mitochondrial, and chloroplast genomes, concatenated together. Mitochondrial and chloroplast genomes have slightly different genetic cores, and the EviAnn software (v2.0.3) can account for that by inputting the names of the organelle contigs with the –mito switch. We extracted the names of the organelle contigs:

```
cat mito_final.fa.PolcaCorrected.fa chloroplast.fasta.PolcaCorrected.fa | ufasta sizes -H |awk '{print $1}' > mito_chlor.txt
```

The command line used for the EviAnn annotation software was:

```
eviann.sh -p proteins.faa -r rnaseq.txt -g BCP_1.0_complete.fa -t 32 -f --mito mito_chlor.txt
```

where proteins.faa was the fasta-formatted file that contained proteins from related conifer species (see manuscript) and rnaseq.txt contained the following:

```
/ccb/salz8-4/alekseyz/BristleconePine/RNA-seq/SRR13823435_1.fastq	/ccb/salz8-4/alekseyz/BristleconePine/RNA-seq/SRR13823435_2.fastq
/ccb/salz8-4/alekseyz/BristleconePine/RNA-seq/SRR13823529_1.fastq	/ccb/salz8-4/alekseyz/BristleconePine/RNA-seq/SRR13823529_2.fastq
/ccb/salz8-4/alekseyz/BristleconePine/RNA-seq/SRR13823538_1.fastq	/ccb/salz8-4/alekseyz/BristleconePine/RNA-seq/SRR13823538_2.fastq
```

/ccb/salz8-4/alekseyz/BristleconePine/RNA-seq/SRR13823539_1.fastq /ccb/salz8-4/alekseyz/BristleconePine/RNA-seq/SRR13823539_2.fastq

/ccb/salz8-4/alekseyz/BristleconePine/RNA-seq/SRR13823540_1.fastq /ccb/salz8-4/alekseyz/BristleconePine/RNA-seq/SRR13823540_2.fastq

/ccb/salz8-4/alekseyz/BristleconePine/RNA-seq/SRR13823620_1.fastq /ccb/salz8-4/alekseyz/BristleconePine/RNA-seq/SRR13823620_2.fastq

/ccb/salz8-4/alekseyz/BristleconePine/RNA-seq/chinese_strobus/SRR13823439_1.fastq
/ccb/salz8-4/alekseyz/BristleconePine/RNA-seq/chinese_strobus/SRR13823439_2.fastq

/ccb/salz8-4/alekseyz/BristleconePine/RNA-seq/chinese_strobus/SRR13823449_1.fastq
/ccb/salz8-4/alekseyz/BristleconePine/RNA-seq/chinese_strobus/SRR13823449_2.fastq

/ccb/salz8-4/alekseyz/BristleconePine/RNA-seq/chinese_strobus/SRR13823452_1.fastq
/ccb/salz8-4/alekseyz/BristleconePine/RNA-seq/chinese_strobus/SRR13823452_2.fastq

/ccb/salz8-4/alekseyz/BristleconePine/RNA-seq/chinese_strobus/SRR13823502_1.fastq
/ccb/salz8-4/alekseyz/BristleconePine/RNA-seq/chinese_strobus/SRR13823502_2.fastq

/ccb/salz8-4/alekseyz/BristleconePine/RNA-seq/chinese_strobus/SRR13823511_1.fastq
/ccb/salz8-4/alekseyz/BristleconePine/RNA-seq/chinese_strobus/SRR13823511_2.fastq

/ccb/salz8-4/alekseyz/BristleconePine/RNA-seq/chinese_strobus/SRR13823519_1.fastq
/ccb/salz8-4/alekseyz/BristleconePine/RNA-seq/chinese_strobus/SRR13823519_2.fastq

/ccb/salz8-4/alekseyz/BristleconePine/RNA-seq/chinese_strobus/SRR13823586_1.fastq
/ccb/salz8-4/alekseyz/BristleconePine/RNA-seq/chinese_strobus/SRR13823586_2.fastq

/ccb/salz8-4/alekseyz/BristleconePine/RNA-seq/chinese_strobus/SRR13823588_1.fastq
/ccb/salz8-4/alekseyz/BristleconePine/RNA-seq/chinese_strobus/SRR13823588_2.fastq

/ccb/salz8-4/alekseyz/BristleconePine/RNA-seq/chinese_strobus/SRR13823621_1.fastq
/ccb/salz8-4/alekseyz/BristleconePine/RNA-seq/chinese_strobus/SRR13823621_2.fastq

/ccb/salz8-4/alekseyz/BristleconePine/RNA-seq/chinese_strobus/SRR13823622_1.fastq
/ccb/salz8-4/alekseyz/BristleconePine/RNA-seq/chinese_strobus/SRR13823622_2.fastq

The annotation output was BCP_1.0_complete.fa.functional_note.pseudo_label.gff, functionally annotated with alignments to UniProt-SwissProt proteins.
