# Supplementary material for: A reference genome sequence for the exceptionally long-lived Great Basin bristlecone pine, Pinus longaeva
Source: G3 (Bethesda). 2026 Mar 17;16(6):jkag064. doi: 10.1093/g3journal/jkag064 (PMC13233319; doi:10.1093/g3journal/jkag064)
Supplement: jkag064_Supplementary_Data [file jkag064_supplementary_data.zip › Table_S1_G3-2026-406665.docx]

**Table S1.** List of 827 plant species from classes Pinopsida and Lycopodiopsida and four species from the angiosperm genus *Populus* whose proteins were used for protein homology evidence for annotation of the bristlecone pine genome with EviAnn. All protein sequences were downloaded from NCBI Genbank.

| *Abies alba*  *Abies amabilis*  *Abies balsamea*  *Abies beshanzuensis*  *Abies borisii-regis*  *Abies bracteata*  *Abies cephalonica*  *Abies cilicica*  *Abies concolor*  *Abies delavayi*  *Abies densa*  *Abies durangensis*  *Abies fargesii*  *Abies firma*  *Abies flinckii*  *Abies forrestii*  *Abies fraseri*  *Abies grandis*  *Abies guatemalensis*  *Abies holophylla*  *Abies homolepis*  *Abies koreana*  *Abies lasiocarpa*  *Abies magnifica*  *Abies mariesii*  *Abies nebrodensis*  *Abies nephrolepis*  *Abies nordmanniana*  *Abies numidica*  *Abies pindrow*  *Abies pinsapo*  *Abies procera*  *Abies recurvata*  *Abies religiosa*  *Abies sachalinensis*  *Abies sibirica*  *Abies spectabilis*  *Abies squamata*  *Abies veitchii*  *Abies vejarii*  *Acmopyle pancheri*  *Acmopyle sahniana*  *Actinostrobus arenarius*  *Actinostrobus pyramidalis*  *Afrocarpus dawei*  *Afrocarpus falcatus*  *Afrocarpus gracilior*  *Afrocarpus mannii*  *Afrocarpus usambarensis*  *Agathis australis*  *Agathis borneensis*  *Agathis corbassonii*  *Agathis dammara*  *Agathis lanceolata*  *Agathis macrophylla*  *Agathis moorei*  *Agathis ovata*  *Agathis robusta*  *Agathis silbae*  *Amentotaxus argotaenia*  *Amentotaxus formosana*  *Amentotaxus poilanei*  *Amentotaxus yunnanensis*  *Araucaria angustifolia*  *Araucaria araucana*  *Araucaria bernieri*  *Araucaria bidwillii*  *Araucaria biramulata*  *Araucaria columnaris*  *Araucaria cunninghamii*  *Araucaria excelsa*  *Araucaria heterophylla*  *Araucaria humboldtensis*  *Araucaria hunsteinii*  *Araucaria laubenfelsii*  *Araucaria luxurians*  *Araucaria montana*  *Araucaria muelleri*  *Araucaria nemorosa*  *Araucaria rulei*  *Araucaria schmidii*  *Araucaria scopulorum*  *Araucaria subulata*  *Athrotaxis cupressoides*  *Athrotaxis laxifolia*  *Athrotaxis selaginoides*  *Austrocedrus chilensis*  *Austrolycopodium magellanicum*  *Austrotaxus spicata*  *Callitris columellaris*  *Callitris gracilis*  *Callitris macleayana*  *Callitris pancheri*  *Callitris preissii*  *Callitris pyramidalis*  *Callitris rhomboidea*  *Callitropsis funebris*  *Callitropsis nootkatensis*  *Callitropsis vietnamensis*  *Calocedrus decurrens*  *Calocedrus formosana*  *Calocedrus macrolepis*  *Cathaya argyrophylla*  *Cedrus atlantica*  *Cedrus brevifolia*  *Cedrus deodara*  *Cedrus libani*  *Cephalotaxus fortunei*  *Cephalotaxus hainanensis*  *Cephalotaxus harringtonia*  *Cephalotaxus mannii*  *Cephalotaxus oliveri*  *Cephalotaxus sinensis*  *Chamaecyparis formosensis*  *Chamaecyparis hodginsii*  *Chamaecyparis lawsoniana*  *Chamaecyparis obtusa*  *Chamaecyparis pisifera*  *Cryptomeria japonica*  *Cunninghamia lanceolata*  *Cupressus atlantica*  *Cupressus cashmeriana*  *Cupressus chengiana*  *Cupressus duclouxiana*  *Cupressus dupreziana*  *Cupressus gigantea*  *Cupressus jiangensis*  *Cupressus sempervirens*  *Cupressus tonkinensis*  *Cupressus torulosa*  *Dacrycarpus cinctus*  *Dacrycarpus compactus*  *Dacrycarpus dacrydioides*  *Dacrycarpus expansus*  *Dacrycarpus imbricatus*  *Dacrycarpus kinabaluensis*  *Dacrycarpus vieillardii*  *Dacrydium araucarioides*  *Dacrydium balansae*  *Dacrydium beccarii*  *Dacrydium comosum*  *Dacrydium cupressinum*  *Dacrydium elatum*  *Dacrydium gracile*  *Dacrydium guillauminii*  *Dacrydium lycopodioides*  *Dacrydium nausoriense*  *Dacrydium nidulum*  *Dacrydium xanthandrum*  *Dendrolycopodium obscurum*  *Diphasiastrum alpinum*  *Diphasiastrum complanatum*  *Diphasiastrum digitatum*  *Diphasiastrum issleri*  *Diphasiastrum madeirense*  *Diphasiastrum montellii*  *Diphasiastrum multispicatum*  *Diphasiastrum nikoense*  *Diphasiastrum sabinifolium*  *Diphasiastrum sitchense*  *Diphasiastrum thyoides*  *Diphasiastrum tristachyum*  *Diphasiastrum veitchii*  *Diphasiastrum wightianum*  *Diphasiastrum zanclophyllum*  *Diphasiastrum zeilleri*  *Diselma archeri*  *Ericetorum pectinatum*  *Falcatifolium falciforme*  *Falcatifolium papuanum*  *Falcatifolium taxoides*  *Fitzroya cupressoides*  *Glyptostrobus pensilis*  *Halocarpus bidwillii*  *Halocarpus kirkii*  *Hesperocyparis arizonica*  *Hesperocyparis bakeri*  *Hesperocyparis benthamii*  *Hesperocyparis forbesii*  *Hesperocyparis glabra*  *Hesperocyparis goveniana*  *Hesperocyparis guadalupensis*  *Hesperocyparis lindleyi*  *Hesperocyparis lusitanica*  *Hesperocyparis macnabiana*  *Hesperocyparis macrocarpa*  *Hesperocyparis montana*  *Hesperocyparis nevadensis*  *Hesperocyparis pygmaea*  *Hesperocyparis sargentii*  *Hesperocyparis stephensonii*  *Huperzia australiana*  *Huperzia lucidula*  *Huperzia selago*  *Huperzia serrata*  *Isoetes abyssinica*  *Isoetes aequinoctialis*  *Isoetes andicola*  *Isoetes andina*  *Isoetes appalachiana*  *Isoetes asiatica*  *Isoetes australis*  *Isoetes azorica*  *Isoetes baodongii*  *Isoetes biafrana*  *Isoetes bolanderi*  *Isoetes boliviensis*  *Isoetes boryana*  *Isoetes butleri*  *Isoetes capensis*  *Isoetes changleensis*  *Isoetes chapmanii*  *Isoetes clavata*  *Isoetes coreana*  *Isoetes coromandelina*  *Isoetes cubana*  *Isoetes delilei*  *Isoetes dixitii*  *Isoetes drummondii*  *Isoetes duriei*  *Isoetes eatonii*  *Isoetes echinospora*  *Isoetes ecuadoriensis*  *Isoetes engelmannii*  *Isoetes flaccida*  *Isoetes gardneriana*  *Isoetes giessii*  *Isoetes graniticola*  *Isoetes gunnii*  *Isoetes gymnocarpa*  *Isoetes hallasanensis*  *Isoetes hawaiiensis*  *Isoetes herzogii*  *Isoetes histrix*  *Isoetes hopei*  *Isoetes howellii*  *Isoetes humilior*  *Isoetes hyemalis*  *Isoetes hypsophila*  *Isoetes jaegeri*  *Isoetes japonica*  *Isoetes jejuensis*  *Isoetes karstenii*  *Isoetes killipii*  *Isoetes kirkii*  *Isoetes lacustris*  *Isoetes laosiensis*  *Isoetes lechleri*  *Isoetes lithophila*  *Isoetes longissima*  *Isoetes louisianensis*  *Isoetes malinverniana*  *Isoetes maritima*  *Isoetes martii*  *Isoetes mattaponica*  *Isoetes melanopoda*  *Isoetes melanospora*  *Isoetes melanotheca*  *Isoetes mexicana*  *Isoetes mississippiensis*  *Isoetes montezumae*  *Isoetes muelleri*  *Isoetes natalensis*  *Isoetes neoguineensis*  *Isoetes nigritiana*  *Isoetes nuttallii*  *Isoetes occidentalis*  *Isoetes olympica*  *Isoetes orcuttii*  *Isoetes organensis*  *Isoetes orientalis*  *Isoetes pallida*  *Isoetes palmeri*  *Isoetes panamensis*  *Isoetes pedersenii*  *Isoetes philippinensis*  *Isoetes piedmontana*  *Isoetes pitotii*  *Isoetes prototypus*  *Isoetes pusilla*  *Isoetes rhodesiana*  *Isoetes riparia*  *Isoetes sampathkumaranii*  *Isoetes saracochensis*  *Isoetes savatieri*  *Isoetes schweinfurthii*  *Isoetes sinensis*  *Isoetes smithii*  *Isoetes spannagelii*  *Isoetes stevensii*  *Isoetes storkii*  *Isoetes taiwanensis*  *Isoetes tegetiformans*  *Isoetes todaroana*  *Isoetes transvaalensis*  *Isoetes tuckermanii*  *Isoetes valida*  *Isoetes virginica*  *Isoetes viridimontana*  *Isoetes weberi*  *Isoetes welwitschii*  *Isoetes wormaldii*  *Isoetes yuhangensis*  *Isoetes yunguiensis*  *Juniperus ashei*  *Juniperus cedrus*  *Juniperus chinensis*  *Juniperus coahuilensis*  *Juniperus communis*  *Juniperus convallium*  *Juniperus drupacea*  *Juniperus durangensis*  *Juniperus erectopatens*  *Juniperus excelsa*  *Juniperus flaccida*  *Juniperus foetidissima*  *Juniperus formosana*  *Juniperus gaussenii*  *Juniperus grandis*  *Juniperus indica*  *Juniperus macrocarpa*  *Juniperus maderensis*  *Juniperus monosperma*  *Juniperus monticola*  *Juniperus morrisonicola*  *Juniperus navicularis*  *Juniperus occidentalis*  *Juniperus osteosperma*  *Juniperus oxycedrus*  *Juniperus phoenicea*  *Juniperus pinchotii*  *Juniperus procera*  *Juniperus przewalskii*  *Juniperus rigida*  *Juniperus sabina*  *Juniperus saltillensis*  *Juniperus saltuaria*  *Juniperus scopulorum*  *Juniperus semiglobosa*  *Juniperus seravschanica*  *Juniperus thurifera*  *Juniperus tibetica*  *Juniperus virginiana*  *Keteleeria davidiana*  *Keteleeria evelyniana*  *Keteleeria fortunei*  *Keteleeria hainanensis*  *Keteleeria pubescens*  *Korallia fruticulosa*  *Lagarostrobos franklinii*  *Larix cajanderi*  *Larix decidua*  *Larix gmelinii*  *Larix griffithii*  *Larix himalaica*  *Larix kaempferi*  *Larix kamtschatica*  *Larix laricina*  *Larix lyallii*  *Larix mastersiana*  *Larix occidentalis*  *Larix potaninii*  *Larix sibirica*  *Larix* sp.  *Larix sukaczewii*  *Lepidothamnus fonkii*  *Lepidothamnus intermedius*  *Lepidothamnus laxifolius*  *Libocedrus austrocaledonica*  *Libocedrus bidwillii*  *Libocedrus plumosa*  *Libocedrus yateensis*  *Lycopodiastrum casuarinoides*  *Lycopodium clavatum*  *Lycopodium deuterodensum*  *Lycopodium lagopus*  *Lycopodium oellgaardii*  *Manoao colensoi*  *Megaloselaginella exaltata*  *Metasequoia glyptostroboides*  *Microbiota decussata*  *Microcachrys tetragona*  *Nageia fleuryi*  *Nageia formosensis*  *Nageia motleyi*  *Nageia nagi*  *Nageia wallichiana*  *Neocallitropsis pancheri*  *Nothotsuga longibracteata*  *Palhinhaea cernua*  *Papuacedrus papuana*  *Parasitaxus usta*  *Pectinopitys exigua*  *Pectinopitys ferruginea*  *Pectinopitys ferruginoides*  *Pectinopitys ladei*  *Pherosphaera fitzgeraldii*  *Phlegmariurus carinatus*  *Phlegmariurus fordii*  *Phlegmariurus myrsinites*  *Phlegmariurus phlegmaria*  *Phlegmariurus squarrosus*  *Phlegmariurus tetrastichus*  *Phyllocladus alpinus*  *Phyllocladus aspleniifolius*  *Phyllocladus hypophyllus*  *Phyllocladus toatoa*  *Phyllocladus trichomanoides*  *Phylloglossum drummondii*  *Picea abies*  *Picea alcoquiana*  *Picea asperata*  *Picea brachytyla*  *Picea breweriana*  *Picea chihuahuana*  *Picea crassifolia*  *Picea engelmannii*  *Picea farreri*  *Picea glauca*  *Picea glehnii*  *Picea jezoensis*  *Picea koraiensis*  *Picea koyamae*  *Picea likiangensis*  *Picea linzhiensis*  *Picea mariana*  *Picea martinezii*  *Picea maximowiczii*  *Picea mexicana*  *Picea meyeri*  *Picea morrisonicola*  *Picea neoveitchii*  *Picea obovata*  *Picea omorika*  *Picea orientalis*  *Picea pungens*  *Picea purpurea*  *Picea retroflexa*  *Picea rubens*  *Picea schrenkiana*  *Picea sitchensis*  *Picea smithiana*  *Picea spinulosa*  *Picea torano*  *Picea wilsonii*  *Pilgerodendron uviferum*  *Pinus albicaulis*  *Pinus aristata*  *Pinus arizonica*  *Pinus armandii*  *Pinus attenuata*  *Pinus ayacahuite*  *Pinus balfouriana*  *Pinus banksiana*  *Pinus bhutanica*  *Pinus brutia*  *Pinus bungeana*  *Pinus canariensis*  *Pinus caribaea*  *Pinus cembra*  *Pinus cembroides*  *Pinus chiapensis*  *Pinus clausa*  *Pinus contorta*  *Pinus coulteri*  *Pinus culminicola*  *Pinus dabeshanensis*  *Pinus dalatensis*  *Pinus densata*  *Pinus densiflora*  *Pinus devoniana*  *Pinus discolor*  *Pinus douglasiana*  *Pinus durangensis*  *Pinus echinata*  *Pinus edulis*  *Pinus elliottii*  *Pinus engelmannii*  *Pinus fenzeliana*  *Pinus flexilis*  *Pinus gerardiana*  *Pinus glabra*  *Pinus greggii*  *Pinus halepensis*  *Pinus hartwegii*  *Pinus heldreichii*  *Pinus herrerae*  *Pinus hwangshanensis*  *Pinus jaliscana*  *Pinus jeffreyi*  *Pinus johannis*  *Pinus koraiensis*  *Pinus krempfii*  *Pinus kwangtungensis*  *Pinus lambertiana*  *Pinus leiophylla*  *Pinus longaeva*  *Pinus massoniana*  *Pinus maximartinezii*  *Pinus maximinoi*  *Pinus merkusii*  *Pinus monophylla*  *Pinus montezumae*  *Pinus monticola*  *Pinus morrisonicola*  *Pinus mugo*  *Pinus muricata*  *Pinus nelsonii*  *Pinus nigra*  *Pinus oocarpa*  *Pinus palustris*  *Pinus parviflora*  *Pinus patula*  *Pinus peuce*  *Pinus pinaster*  *Pinus pinceana*  *Pinus pinea*  *Pinus ponderosa*  *Pinus pseudostrobus*  *Pinus pumila*  *Pinus quadrifolia*  *Pinus radiata*  *Pinus remota*  *Pinus resinosa*  *Pinus rigida*  *Pinus roxburghii*  *Pinus rzedowskii*  *Pinus sabiniana*  *Pinus sibirica*  *Pinus squamata*  *Pinus strobiformis*  *Pinus strobus*  *Pinus sylvestris*  *Pinus tabuliformis*  *Pinus taeda*  *Pinus taiwanensis*  *Pinus teocote*  *Pinus thunbergii*  *Pinus torreyana*  *Pinus virginiana*  *Pinus wallichiana*  *Pinus wangii*  *Pinus yecorensis*  *Pinus yunnanensis*  *Platycladus orientalis*  *Podocarpus acutifolius*  *Podocarpus affinis*  *Podocarpus angustifolius*  *Podocarpus annamiensis*  *Podocarpus archboldii*  *Podocarpus aristulatus*  *Podocarpus atjehensis*  *Podocarpus beecherae*  *Podocarpus bracteatus*  *Podocarpus brasiliensis*  *Podocarpus brassii*  *Podocarpus brevifolius*  *Podocarpus capuronii*  *Podocarpus celatus*  *Podocarpus chingianus*  *Podocarpus coriaceus*  *Podocarpus costalis*  *Podocarpus crassigemmis*  *Podocarpus cunninghamii*  *Podocarpus decipiens*  *Podocarpus decumbens*  *Podocarpus deflexus*  *Podocarpus degeneri*  *Podocarpus dispermus*  *Podocarpus drouynianus*  *Podocarpus elatus*  *Podocarpus elongatus*  *Podocarpus fasciculus*  *Podocarpus forrestii*  *Podocarpus gibbsiae*  *Podocarpus gnidioides*  *Podocarpus grayae*  *Podocarpus guatemalensis*  *Podocarpus hallii*  *Podocarpus henkelii*  *Podocarpus insularis*  *Podocarpus lambertii*  *Podocarpus latifolius*  *Podocarpus lawrencei*  *Podocarpus ledermannii*  *Podocarpus longifoliolatus*  *Podocarpus lucienii*  *Podocarpus macrophyllus*  *Podocarpus madagascariensis*  *Podocarpus magnifolius*  *Podocarpus matudae*  *Podocarpus milanjianus*  *Podocarpus nakaii*  *Podocarpus neriifolius*  *Podocarpus nivalis*  *Podocarpus novae-caledoniae*  *Podocarpus nubigenus*  *Podocarpus oleifolius*  *Podocarpus pallidus*  *Podocarpus parlatorei*  *Podocarpus philippinensis*  *Podocarpus pilgeri*  *Podocarpus polyspermus*  *Podocarpus polystachyus*  *Podocarpus pseudobracteatus*  *Podocarpus purdieanus*  *Podocarpus ramosii*  *Podocarpus rostratus*  *Podocarpus rubens*  *Podocarpus rumphii*  *Podocarpus rusbyi*  *Podocarpus salignus*  *Podocarpus salomoniensis*  *Podocarpus sellowii*  *Podocarpus smithii*  *Podocarpus spathoides*  *Podocarpus spinulosus*  *Podocarpus sprucei*  *Podocarpus subtropicalis*  *Podocarpus sylvestris*  *Podocarpus teysmannii*  *Podocarpus thailandensis*  *Podocarpus totara*  *Podocarpus transiens*  *Podocarpus trinitensis*  *Podocarpus urbanii*  *Populus alba*  *Populus deltoides*  *Populus euphratica*  *Populus tomentosa*  *Prumnopitys amara*  *Prumnopitys andina*  *Prumnopitys ferruginoides*  *Prumnopitys ladei*  *Prumnopitys montana*  *Prumnopitys taxifolia*  *Pseudolarix amabilis*  *Pseudolycopodiella caroliniana*  *Pseudotaxus chienii*  *Pseudotsuga forrestii*  *Pseudotsuga japonica*  *Pseudotsuga macrocarpa*  *Pseudotsuga menziesii*  *Pseudotsuga sinensis*  *Retrophyllum comptonii*  *Retrophyllum minus*  *Retrophyllum rospigliosii*  *Retrophyllum vitiense*  *Saxegothaea conspicua*  *Sciadopitys verticillata*  *Selaginella acanthonota*  *Selaginella albocincta*  *Selaginella alutacea*  *Selaginella anceps*  *Selaginella apoda*  *Selaginella argentea*  *Selaginella arizonica*  *Selaginella asperula*  *Selaginella australiensis*  *Selaginella balansae*  *Selaginella bamleri*  *Selaginella behrmanniana*  *Selaginella biformis*  *Selaginella bisulcata*  *Selaginella bluuensis*  *Selaginella braunii*  *Selaginella brevipes*  *Selaginella breynioides*  *Selaginella brooksii*  *Selaginella burkei*  *Selaginella cathedrifolia*  *Selaginella caudata*  *Selaginella chrysocaulos*  *Selaginella chuweimingii*  *Selaginella cladorrhizans*  *Selaginella conduplicata*  *Selaginella crassipes*  *Selaginella davidii*  *Selaginella delicatula*  *Selaginella densa*  *Selaginella denticulata*  *Selaginella distans*  *Selaginella doederleinii*  *Selaginella douglasii*  *Selaginella dregei*  *Selaginella echinata*  *Selaginella effusa*  *Selaginella erythropus*  *Selaginella eurynota*  *Selaginella falcata*  *Selaginella frondosa*  *Selaginella geniculata*  *Selaginella gracillima*  *Selaginella griffithii*  *Selaginella haematodes*  *Selaginella harrisii*  *Selaginella helvetica*  *Selaginella hieronymiana*  *Selaginella hoffmannii*  *Selaginella hordeiformis*  *Selaginella huehuetenangensis*  *Selaginella ingens*  *Selaginella intermedia*  *Selaginella involvens*  *Selaginella kerstingii*  *Selaginella kochii*  *Selaginella kraussiana*  *Selaginella labordei*  *Selaginella landii*  *Selaginella laxa*  *Selaginella laxistrobila*  *Selaginella lechleri*  *Selaginella lepidophylla*  *Selaginella lingulata*  *Selaginella longiaristata*  *Selaginella lyallii*  *Selaginella lychnuchus*  *Selaginella mairei*  *Selaginella marginata*  *Selaginella martensii*  *Selaginella mayeri*  *Selaginella miniatospora*  *Selaginella moellendorffii*  *Selaginella moratii*  *Selaginella moritziana*  *Selaginella muscosa*  *Selaginella mutica*  *Selaginella myosurus*  *Selaginella nana*  *Selaginella neocaledonica*  *Selaginella nipponica*  *Selaginella nothohybrida*  *Selaginella novae-hollandiae*  *Selaginella oaxacana*  *Selaginella ornata*  *Selaginella pallescens*  *Selaginella pallidissima*  *Selaginella parkeri*  *Selaginella peruviana*  *Selaginella pervillei*  *Selaginella plana*  *Selaginella potaroensis*  *Selaginella producta*  *Selaginella pulcherrima*  *Selaginella pulvinata*  *Selaginella radiata*  *Selaginella rechingeri*  *Selaginella reflexa*  *Selaginella reineckei*  *Selaginella remotifolia*  *Selaginella revoluta*  *Selaginella roxburghii*  *Selaginella rupincola*  *Selaginella sandwithii*  *Selaginella sanguinolenta*  *Selaginella sechellarum*  *Selaginella selaginoides*  *Selaginella sellowii*  *Selaginella serpens*  *Selaginella sertata*  *Selaginella siamensis*  *Selaginella sibirica*  *Selaginella silvestris*  *Selaginella sinensis*  *Selaginella stipulata*  *Selaginella tamariscina*  *Selaginella tortipila*  *Selaginella trachyphylla*  *Selaginella uliginosa*  *Selaginella uncinata*  *Selaginella underwoodii*  *Selaginella vaginata*  *Selaginella vardei*  *Selaginella versicolor*  *Selaginella viridangula*  *Selaginella viticulosa*  *Selaginella vogelii*  *Selaginella wallacei*  *Selaginella wallichii*  *Selaginella whitmeei*  *Selaginella wightii*  *Selaginella willdenowii*  *Selaginella yemensis*  *Sequoiadendron giganteum*  *Sequoia sempervirens*  *Spinulum annotinum*  *Sundacarpus amarus*  *Taiwania cryptomerioides*  *Taxodium distichum*  *Taxodium mucronatum*  *Taxus baccata*  *Taxus brevifolia*  *Taxus calcicola*  *Taxus canadensis*  *Taxus celebica*  *Taxus chinensis*  *Taxus contorta*  *Taxus cuspidata*  *Taxus floridana*  *Taxus florinii*  *Taxus fuana*  *Taxus globosa*  *Taxus mairei*  *Taxus phytonii*  *Taxus sumatrana*  *Taxus wallichiana*  *Tetraclinis articulata*  *Thuja koraiensis*  *Thuja occidentalis*  *Thuja plicata*  *Thuja standishii*  *Thuja sutchuenensis*  *Thujopsis dolabrata*  *Torreya californica*  *Torreya fargesii*  *Torreya grandis*  *Torreya jackii*  *Torreya nucifera*  *Torreya taxifolia*  *Tsuga canadensis*  *Tsuga caroliniana*  *Tsuga chinensis*  *Tsuga diversifolia*  *Tsuga dumosa*  *Tsuga heterophylla*  *Tsuga mertensiana*  *Tsuga sieboldii*  *Tsuga ulleungensis*  *Widdringtonia cedarbergensis*  *Widdringtonia nodiflora*  *Wollemia nobilis* |
| --- |
